# Licit Substance Use and Premenstrual Syndrome Symptom Severity in Female College Students

**DOI:** 10.1089/whr.2021.0117

**Published:** 2022-05-02

**Authors:** Kathryn Polak, Pamela Nora, Bridget Perry, Caitlin Martin, Pam Dillon, Leroy Thacker, Sarah Nance, Susan Kornstein, Dace Svikis

**Affiliations:** ^1^ Department of Psychology, Virginia Commonwealth University, Richmond, Virginia, USA.; ^2^ Private Practice, Fairfield, Connecticut, USA.; ^3^ Department of Obstetrics and Gynecology and Institute for Drug and Alcohol Studies, Virginia Commonwealth University, Richmond, Virginia, USA.; ^4^ Center for Clinical and Translational Research, Virginia Commonwealth University, Richmond, Virginia, USA.; ^5^ School of Nursing, Virginia Commonwealth University, Richmond, Virginia, USA.; ^6^ Institute for Women's Health, Virginia Commonwealth University, Richmond, Virginia, USA.; ^7^ Department of Psychiatry, Virginia Commonwealth University, Richmond, Virginia, USA.

**Keywords:** college students, caffeine, alcohol, tobacco, premenstrual syndrome

## Abstract

**Introduction:**

Premenstrual syndrome (PMS) affects the majority of women and is characterized by physical, behavioral, and mood symptoms, which can have a profound impact on quality of life. PMS symptoms have also been linked to licit substance use. This study examined the relationships between daily/problem use (DPU) of caffeine (Caf^+^), alcohol (Alc^+^), and tobacco (Cig^+^) and PMS symptomology in a sample of college women.

**Methods:**

Participants (*N* = 196) completed an anonymous one-time health survey. Demographic, PMS symptomatology, and DPU of licit substance variables were examined. Independent *t*-tests compared PMS symptom scores in women with and without Caf^+^, Cig^+^, and Alc^+^ use. One-way analysis of variances examined the associations between PMS symptom severity and number of DPU-positive substances.

**Results:**

PMS subscale severity (pain [*F*(2,190) = 4.47, *p* = 0.013], affective [*F*(2,192) = 8.21, *p* < 0.001], and water retention [*F*(2,191) = 13.37, *p* < 0.001]) and total PMS symptom severity [*F*(2,189) = 10.22, *p* < 0.001] showed a dose response effect, with the number of licit substances with DPU significantly associated with PMS symptom severity.

**Conclusions:**

This study findings provide important new information about the relationship between PMS symptoms and at-risk substance use. These are cross-sectional data, however, and affirm a need for longitudinal research to better understand the associations, with a focus on potential benefits of education and intervention.

## Introduction

The vast majority (∼90%) of women of childbearing age experience at least some premenstrual syndrome (PMS) symptoms.^1^ PMS is characterized by physical (e.g., breast tenderness, headache, and bloating), behavioral (e.g., sleep disturbance, appetite fluctuation, and diminished interest), and mood (e.g., anxiety, mood swings, and irritability) symptoms occurring in the luteal phase of the menstrual cycle and lasting an average of 6 days each cycle.^2^ In the United States, 20%–40% of women of childbearing age report moderately severe PMS.^3^

PMS symptoms can have a profound impact on quality of life. One-fifth (20%) of women with PMS symptoms report distress due to their PMS symptoms, ∼3%–8% experience functional impairment in interpersonal or occupational domains,^3^ and 2%–8% meet DSM-5 criteria for premenstrual dysphoric disorder (PMDD),^4–6^ PMDD's health-related quality of life burden is similar to that of osteoarthritis and rheumatoid arthritis and higher than that for chronic back pain.^7^

Furthermore, compared with those reporting no/mild PMS symptoms, women with moderate-to-severe PMS or PMDD report decreased health-related quality of life, less participation in hobbies/social activities, and more relationship disruption.^8^ Although effective treatments are available (see Maharaj & Trevino, 2015, for a full review of treatment options),^9^ the majority of women with premenstrual disorders do not receive treatment. This occurs because many treatment options are relatively new and the effects of PMS/PMDD are not widely known among both professionals and the general public.^5,6^

The medical and psychosocial costs associated with PMS/PMDD are substantial.^10^ Compared with women with mild/no PMS, those with severe PMS/PMDD have an 80% increased likelihood of >10 physician visits in 2 years and are twice as likely to have >$500 in health care costs.^11^ For PMDD in the US, the disability-adjusted life years is about 14 million.^6^ Furthermore, moderate-to-severe PMS/PMDD is associated with greater work absenteeism, compromised work productivity, and impaired daily life activities compared with mild/no PMS.^12^

Research on the relationship between alcohol use and PMS/PMDD symptom severity has been mixed. A number of studies have found a positive relationship between alcohol use and PMS/PMDD symptom severity across diverse samples that range from U.S. college students,^13^ community-based samples,^14^ and medical patients^15,16^ to non-U.S. samples.^17^ A recent metanalysis found alcohol use was associated with a moderate increase in risk of PMS.^18^ Others, however, found no significant associations between alcohol use and PMS symptomatology.^19,20^ Similarly, Parazzini et al. found that heavy alcohol use decreased the risk of dysmenorrhea.^21^

In contrast, caffeine use has been linked to specific PMS symptoms, in particular, premenstrual irritability and insomnia.^22^ In a sample of Saudi Arabian women, PMS symptoms were positively associated with coffee use.^23^ Similarly, Rossignol and Bonnlander found a dose-dependent relationship between caffeine use and PMS symptom prevalence and severity.^24^

For tobacco, much research has focused on smoking cessation and its effect on PMS symptoms; however, few studies have proposed a link between tobacco use and symptomology. In Japan, Sakai et al. found regular smokers had more severe PMS symptoms than nonregular smokers, and scores on a measure of premenstrual symptom severity were positively correlated with motives for smoking and nicotine dependence severity.^25^ Current cigarette smoking, even after adjusting for other factors, was associated with an increased risk of every menstrual symptom and cycle disorder among women in the U.S. navy.^19^ Correspondingly, in a case–control study from Italy, the relative risk of dysmenorrhea increased with the duration of smoking and the number of cigarettes smoked per day.^21^

This study afforded a unique opportunity to look separately at relationships between daily/problem use (DPU) of caffeine, alcohol, and tobacco and PMS symptom severity in a sample of college women. We hypothesized that for each substance, daily use or problems would be linked with higher PMS symptom and subscale severity. In addition, we hypothesized that there would be an association between number of licit substances used daily/problematically and individual as well as PMS subscale scores. Understanding the relationship between substance use and PMS symptom severity is the first step in improving targeted interventions for substance use and PMS. Treatments for substance use may be potential targets to also reduce symptomatology of PMS and its effects on quality of life of affected women.

## Materials and Methods

### Participants

Participants were *N* = 196 female undergraduate psychology students who completed an anonymous health survey for course credit at an urban university.

### Procedure and measures

Students were recruited from undergraduate psychology courses. In an effort to limit reactivity, participants were told the study's purpose was to investigate the relationship between stress, coping, and women's health. Women providing verbal consent subsequently completed a 45–60-minute self-administered paper-and-pencil assessment battery. Research assistants were available to answer questions and participants were compensated for their time and effort with class credit. The research was reviewed and approved by the university institutional review board (IRB).

The health survey for the original dissertation study of PMS symptoms and alcohol use focused on a variety of psychosocial health domains (see Perry et al. for detailed summary).^26^ This study looked at demographic (age, race, and year in school), PMS symptomatology, and licit substance use variables.

#### Premenstrual symptomatology

PMS symptoms were assessed using the 10-item Shortened Premenstrual Assessment Form (SPAF), which has been shown to be reliable and valid.^27^ The SPAF asks participants to rate the intensity of premenstrual symptoms during the 7 days before their last menstrual cycle on a scale from 1 (not present or no change from usual) to 6 (extreme change, perhaps noticeable even to casual acquaintances). In addition, three subscale scores (water retention, affective, and pain severity) and total PMS symptom severity scores were calculated (Table 1).

**Table 1.** Shortened Premenstrual Assessment Form Item and Subscale Means (Standard Deviations) with Corresponding ***t*-Test *p*-Values for Caf^+^, Cig^+^, and Alc^+^ Use**

PMS symptom subscales and individual items	Caffeine			Cigarettes			Alcohol		
	Caf^+^ mean (***SD***)	Caf^−^ mean (***SD***)	***p***	Cig^+^ mean (***SD***)	Cig^−^ mean (***SD***)	***p***	Alc^+^ mean (***SD***)	Alc^−^ mean (***SD***)	***p***
Pain subscale	10.48 (3.99)	8.85 (3.91)	**0.03**	10.18 (4.36)	8.94 (3.88)	0.12	10.82 (3.71)	8.62 (3.85)	**0.002**
Have pain, tenderness, enlargement, or swelling breasts.	3.81 (1.67)	3.11 (1.59)	**0.02**	3.41 (1.74)	3.19 (1.61)	0.48	3.73 (1.55)	3.08 (1.59)	**0.02**
Tend to have headaches, joint or muscle pain, or stiffness.	3.19 (1.68)	2.84 (1.53)	0.23	3.29 (1.56)	2.84 (1.54)	0.15	3.54 (1.54)	2.69 (1.49)	**0.002**
Have relatively steady abdominal heaviness, discomfort, or pain.	3.59 (1.64)	2.89 (1.62)	**0.03**	3.62 (1.66)	2.90 (1.62)	**0.03**	3.65 (1.63)	2.84 (1.61)	**0.005**
Affective subscale	14.16 (5.72)	11.89 (5.66)	**0.04**	13.90 (5.73)	12.01 (5.67)	0.10	15.45 (5.59)	11.12 (5.49)	**<0.001**
Feel that I just “can't cope” or am overwhelmed by ordinary demands.	3.19 (1.69)	2.51 (1.49)	**0.02**	3.10 (1.54)	2.54 (1.52)	0.06	3.45 (1.60)	2.37 (1.47)	**<0.001**
Feel under stress.	3.81 (1.58)	3.26 (1.62)	0.07	3.83 (1.65)	3.27 (1.61)	0.09	4.13 (1.59)	3.07 (1.58)	**<0.001**
Have outbursts of “irritability” or bad temper.	3.66 (1.49)	3.05 (1.73)	0.06	3.45 (1.57)	3.10 (1.72)	0.31	3.93 (1.69)	2.83 (1.65)	**<0.001**
Feel sad or blue.	3.50 (1.52)	3.08 (1.57)	0.16	3.52 (1.55)	3.09 (1.56)	0.18	3.95 (1.40)	2.85 (1.52)	**<0.001**
Water retention subscale	9.47 (4.02)	7.65 (3.72)	**0.01**	9.64 (4.51)	7.67 (3.65)	**0.03**	10.73 (4.26)	7.25 (3.33)	**<0.001**
Have weight gain.	3.00 (1.57)	2.49 (1.45)	0.07	3.00 (1.59)	2.51 (1.45)	0.10	3.63 (1.51)	2.35 (1.38)	**<0.001**
Have edema, swelling, puffiness, or water retention.	2.75 (1.57)	2.14 (1.37)	**0.02**	3.04 (1.62)	2.12 (1.34)	**0.001**	3.13 (1.62)	1.99 (1.24)	**<0.001**
Feel bloated.	3.72 (1.63)	3.02 (1.53)	**0.02**	3.62 (1.76)	3.04 (1.52)	0.06	3.98 (1.64)	2.92 (1.45)	**<0.001**

Bold indicates significant *p*-values.

PMS, premenstrual syndrome; SD, standard deviation.

This study created the following substance use variables, with cutoffs for each item chosen as indicators of risk for development of a substance use disorder:

#### Daily coffee use

“Do you drink caffeinated coffee on a daily basis?” with response options: “Yes” (Caf^+^; *n* = 29) or “No” (Caf^−^; *n* = 167).

#### Daily cigarette smoking

National household survey on drug abuse^28^ item dichotomized into daily current smoker (Cig^+^; *n* = 27) or nondaily smoker (Cig^−^; *n* = 169) groups.

#### Harmful/hazardous alcohol use

Scores on the alcohol use disorders identification test (AUDIT) (range 0–40), a reliable and valid screening tool for identifying persons at risk for problem drinking, were dichotomized using a standardized cutoff into potentially harmful/hazardous (AUDIT ≥8; Alc^+^; *n* = 40) and nonproblem (AUDIT ≤7; Alc^−^; *n* = 156) alcohol use groups.^29^

#### Number of substances positive for DPU

Caf^+^, Cig^+^, and Alc^+^ (yes/no) were summed to create an index of risk (range: 0 to 3 substances) from low (none endorsed; *n* = 129) to moderate (1 substance; *n* = 44) to considerable [2 (*n* = 17) or 3 (*n* = 6) substances; combined due to small *n'*s].

### Data analysis

Independent *t*-tests were used to compare individual PMS symptom scores and mean SPAF subscale scores in women with and without Caf^+^, Cig^+^, and Alc^+^ use. One-way analysis of variances (ANOVAs) were used to examine the associations between PMS symptom severity and the number of substances positive for DPU for each of three subscales as well as total PMS symptom severity. Multiple comparisons of all means were carried out using a *post hoc* Tukey–Kramer honestly significant difference (HSD) test (*α* = 0.05). All analyses were completed using SPSS version 22.

## Results

### Sample demographics

Study participants were female (100%); more than half were Caucasian (57.9%) and approximately one-fourth were African American (22.6%). On average, participants were 19 years old (*M*, mean [standard deviation, *SD*] = 19.30 [2.34] years; range 18 to 34 years). Nearly three-fourths of participants were freshmen (70.8%), with another 17.7% in their sophomore year.

### PMS symptom severity by daily/problem substance use group

Mean PMS symptom severity scores for the 10 SPAF items and subscales and *p*-values for *t*-test comparisons for Caf^+^ (*n* = 29; 14.8%), Cig^+^ (*n* = 27; 13.8%), and Alc^+^ (*n* = 40; 20.4%) are summarized in Table 1. Significant differences in symptom severity were found, with higher PMS scores on 5 of 10 symptoms for Caf^+^, 2 of 10 symptoms for Cig^+^, and 10 of 10 symptoms for Alc^+^. In all cases, the group positive for DPU reported greater average symptom severity when compared with the no DPU group.

### Total PMS symptom severity

The same relationship was observed for total PMS symptom severity. A one-way ANOVA showed that the number of substances positive for DPU was significantly associated with increased total PMS symptom severity, [*F*(2,189) = 10.22, *p* < 0.001]. Tukey's HSD *post hoc* tests found total PMS symptom severity scores were significantly lower among those with no DPU (*M* = 26.47, *SD* = 10.66) as compared with both those positive for one substance (*M* = 32.67, *SD* = 12.33) and those positive for two to three substances (*M* = 36.45, *SD* = 12.83).

### PMS subscale scores by number of substances positive for DPU

**FIG. 1.**
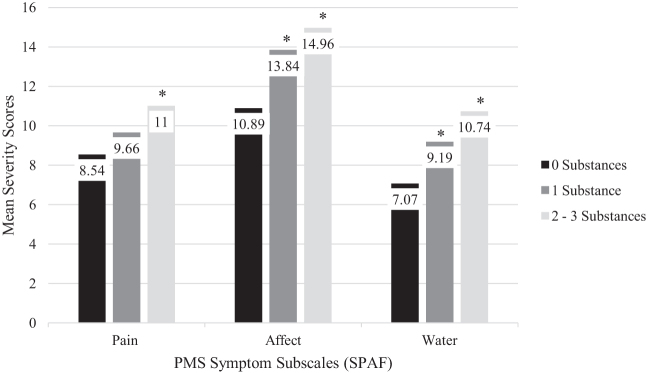
PMS symptom severity and number of licit substances positive for DPU. *Significantly higher scores (*p* < 0.05) compared with those reporting 0 substances positive for DPU. DPU, daily/problem use; PMS, premenstrual syndrome.

As shown in Figure 1, PMS subscale severity scores for pain [*F*(2,190) = 4.47, *p* = 0.013], affective symptoms [*F*(2,192) = 8.21, *p* < 0.001], and water retention [*F*(2,191) = 13.37, *p* < 0.001]) increased as a function of the number of substances positive for DPU.

## Discussion

### Principal findings

This study is unique in its focus on the relationship between PMS symptomatology and cumulative DPU of caffeine (coffee), tobacco, and alcohol in college females. We found Caf^+^, Cig^+^, and Alc^+^ use was each associated with greater PMS symptom severity, symptom subscale scores, and total severity scores in college women. Furthermore, we found PMS subscale and total scores increased as the number of licit substances positive for DPU also increased.

Our study is among the first to concurrently explore all three licit substances separately as well as in combination. By focusing on specific PMS symptoms, overall severity, and symptom domains as well as the relationship between these licit substances, our findings provide finer detail regarding the nature of the previously identified relationships between PMS symptoms and DPU of individual licit substances.

For alcohol, this study findings of a positive relationship between PMS symptoms (across pain, affective, and water retention subscales) and heavy/problem drinking were consistent with the literature, with reports of associations between gynecological issues, mental health concerns, and problem drinking.^18,30,31^ Previous research has predominantly focused on alcohol use and PMS symptoms, with many studies reporting associations between PMS symptoms and heavy/problem drinking similar to those of our study.^14,16,19,32^

These findings must be interpreted cautiously given that PMS and problem drinking share symptoms in common, making it difficult to discern causality. For example, headaches, mood, and behavioral changes (e.g., increased irritability, depression, and altered sleep patterns), as well as decreased focus are symptoms common to both PMS and alcohol use.^33^ Our findings also build upon previous research, which found women may cope with affective symptoms of PMS by drinking.^26^

This study found associations between Caf^+^ and Cig^+^ and increased PMS symptomatology. Compared with women with Caf^−^, those with Caf^+^ reported significantly more pain, affective, and water retention symptoms. Similarly, the Cig^+^ group reported significantly more water retention symptoms, with trends for increased pain and affective symptoms compared with Cig^−^ counterparts. Relationships between these two licit substances and PMS symptomatology have historically received much less attention. For caffeine, this study findings are consistent with limited reports of an association between increased PMS symptoms and greater caffeine use.^23,24^

There is some evidence that caffeine may impact emotional fluctuations linked with the menstrual cycle.^34^ Such mood changes may result from interactive effects of caffeine and reproductive steroid hormones and adenosine.^35^ Rossignol et al. found that increased caffeine intake is associated with higher PMS symptom severity and it may be used to self-medicate for PMS symptoms.^36^ Although there have been reports of associations between caffeine use and breast pain and bloating, no direct relationships have been found.^37,38^

For tobacco, previous research found a relationship between PMS symptom severity and both any tobacco use and nicotine dependency.^29^ In addition to Latthe et al., our study is one of the first to look at PMS symptom severity as a function of daily tobacco use.^39^ This is particularly important given the fact that nicotine use and cessation have been found to vary as a function of the menstrual cycle, potentially as a result of the impact of sex hormones on the dopamine reward pathway.^35^ Women with increased anger and cravings who made quit attempts during the follicular phase were more likely to relapse to smoking at 14 days.^40^

In addition, both variability in symptoms and craving a cigarette have been associated with relapse.^41^ As a result, it has been suggested that cessation efforts should be timed to coincide with specific menstrual phases (e.g., quit dates avoiding the luteal phase, which is associated with increased smoking and withdrawal symptoms).^42^ Furthermore, although quantity of daily cigarette use was not related to menstrual phase, severity of premenstrual symptoms, mood, and tobacco withdrawal symptoms all were higher during the luteal than during the follicular phase.^26,43^

We found that pain subscale scores increased as a function of the number of licit substances positive for DPU. Past findings on the relationship between substance use and pain from menses and cycle disorders have been mixed. For example, Parazzini et al. found an association between alcohol consumption and endometriosis, whereas Latthe et al. found no relationship between alcohol use and dysmenorrhea.^39,44^ These inconclusive findings may reflect the lack of incorporating important biopsychosocial and psychological factors related to pain outcomes, such as catastrophizing,^45^ into analyses. Thus, our findings support this need for further work to disentangle the association between substance use and menstrual pain disorders.

Our finding of a dose–response relationship between the number of DPU-positive licit substances and PMS symptom severity could lend further support for the hypothesis that these substances potentially are being used to self-medicate for PMS symptoms. Alternatively, the dose–response relationship may result from the fact that these substances can potentially exacerbate PMS symptom severity.^43^ Lastly, healthy lifestyle characteristics (e.g., healthy eating, decreased stress, and exercise) have been linked to decreased PMS symptom severity,^46,47^ possibly indicating our results could be attributable to underlying general health. As our study is cross-sectional in design, we cannot posit a causal relationship between the number of substances positive for DPU and PMS symptom severity.

### Limitations

There are several limitations to this study. First, the sample was restricted to college females, and results may not generalize to the broader female population. Nonetheless, this is an important subgroup, which has been linked with increased substance use and problems.^48^ Second, PMS symptom severity was assessed retrospectively, an approach found to be less valid than prospective symptom assessment.^49^ Nonetheless, a standardized measure (SPAF) was used, which provides detailed information about specific PMS symptoms and subscales.^27^ Third, the survey did not assess how PMS symptoms impacted quality of life, a standard measure in clinical screenings that informs a diagnosis, and there were no questions about PMDD or baseline symptoms when they were not experiencing PMS.

However, our study provides both a window into PMS symptomatology and support to study these relationships using noncross-sectional data. Fourth, although standardized measures were used to assess alcohol and cigarette use, caffeine use was assessed with a single item on daily coffee use. As a result, we potentially did not capture all regular caffeine users or important variability in caffeine use. Nonetheless, coffee has a high caffeine content and is widely used as a proxy for caffeine use.^50–52^ Lastly, although we did not distinguish between current and past smokers or those who smoke differing amounts in our analyses, frequency of cigarette use (i.e., daily use) is a reliable metric of development of dependence.^53^

### Future research

This study extends previous findings on the relationship between risky alcohol use and PMS symptom severity and highlights an important area of women's health receiving less attention of late. It also provides important new information about the relationship between PMS symptom severity and DPU-positive caffeine and cigarette use as well as the combination of all three licit substances. These cross-sectional data warrant further study to determine whether they are causal or correlational, with a focus on potential benefits of education and intervention.

These studies should also include examination of other lifestyle risk factors, especially since PMS has been linked to reduced quality of sleep, psychiatric morbidity, cholesterol,^17^ and illicit drug use.^54^ Future investigations should examine variables that have been shown to impact the association between premenstrual symptomatology and substance use, such as family history of alcoholism.^55^ In addition, further investigation is needed into the relationship between PMS and caffeine and tobacco, including variation in use, problematic use, and development of caffeine and tobacco use disorders.

For instance, since it is currently recommended that caffeine use be restricted among PMS symptomatic women,^22^ the effect of caffeine restriction on PMS symptomatology should be examined to determine whether this is actually beneficial. Lastly, mobile phone apps have recently been shown to facilitate improved menstrual cycle tracking,^56^ and future studies could explore the use of technology in monitoring and identifying PMS symptom severity.

## Abbreviations Used

ANOVA: analysis of variance
AUDIT: alcohol use disorders identification test
DPU: daily/problem use
HSD: honestly significant difference
IRB: institutional review board
PMDD: premenstrual dysphoric disorder
PMS: premenstrual syndrome
SD: standard deviation
SPAF: Shortened Premenstrual Assessment Form
